# Sex difference in disease burden of inflammatory arthritis patients treated with tumor necrosis factor inhibitors as part of standard care

**DOI:** 10.1371/journal.pone.0266816

**Published:** 2022-05-05

**Authors:** Brigitte Michelsen, Kristine Thomassen Berget, Jon Håvard Loge, Arthur Kavanaugh, Glenn Haugeberg

**Affiliations:** 1 Division of Rheumatology, Department of Medicine, Sørlandet Hospital Kristiansand, Kristiansand, Norway; 2 Department of Clinical Immunology and Transfusion Medicine, Sørlandet Hospital Kristiansand, Kristiansand, Norway; 3 Faculty of Medicine, University of Oslo, Oslo, Norway; 4 Division of Rheumatology, Allergy, Immunology, University of California San Diego, San Diego, California, United States of America; 5 Faculty of Medicine and Health Sciences, Department of Neuromedicine and Movement Science, NTNU, Norwegian University of Science and Technology, Trondheim, Norway; SERGAS and IDIS, SPAIN

## Abstract

**Objective:**

Knowledge is needed on the total disease burden across the sexes in inflammatory arthritis (IA). We aimed to compare disease burden, including a broad range of health aspects, across men and women with IA treated with tumor necrosis factor inhibitors (TNFi).

**Methods:**

Adult outpatients with IA (rheumatoid arthritis, ankylosing spondylitis, psoriatic arthritis) were included as part of standard care. Patient-reported outcomes, disease activity, TNFi trough levels, calprotectin, Work Productivity and Activity Impairment, comorbidities and cardiovascular risk profile were assessed. Unadjusted comparisons across sexes were done with independent t-test, Mann-Whitney U-test and X^2^-test and adjusted analyses with General Linear Models and logistic/ordinal logistic regression.

**Results:**

A total of 305 IA patients were included (167 men, 138 women). A significantly lower proportion of women (45%) than men (59%) were in remission according to disease-specific composite scores (p = 0.02). Women had significantly worse scores on pain, joint pain, fatigue, enthesitis, Health Assessment Questionnaire and Short Form (SF)-36 vitality and social functioning (all p≤0.04). Both sexes had worse SF-36 scale scores than the general population. Women reported more absenteeism (work time missed) and activity impairment. TNFi trough levels, neutralizing antibodies and calprotectin were similar across sexes. A similar total number of comorbidities was seen. Self-reported hypothyroidism was more frequent in women. Men had higher 10-year calculated risk of fatal cardiovascular events.

**Conclusion:**

Important differences in disease burden between men and women were seen. More attention to sex differences in the follow-up of IA patients is warranted.

## Introduction

Rheumatoid arthritis (RA), psoriatic arthritis (PsA) and ankylosing spondylitis (AS) are the most prevalent of the inflammatory arthritides (IA) [1,2]. The treat-to-target approach, together with the biologic disease-modifying anti-rheumatic drugs (bDMARDs), have led to improved outcomes for patients with IA during the last two decades [3]. The most common bDMARDs used for treatment of IA are the Tumor Necrosis Factor inhibitors (TNFi), although several other treatment options (e.g. targeted synthetic DMARDs) have been introduced in recent years [4,5].

Patients with IA may suffer from pain in e.g. joints and spine, fatigue, reduced Health-Related Quality of Life (HRQoL), functional loss and disability [6]. Studies have shown differences not only in disease characteristics between men and women with IA, but also in treatment effectiveness [7–10]. Women tend to respond less to treatment and to have poorer subjective health, including higher levels of pain and worse health-related quality of life (HRQoL) than men [7–13].

Although sex differences in characteristics and treatment effectiveness in IA are known, there is a lack of observational studies addressing the overall disease burden—including a broad range of health aspects—across the sexes. Further, there is a lack of comprehensive knowledge on the different disease manifestations in women [14].

Hence, in this real-life study we aimed to compare disease burden across men and women with IA treated with TNFi, including patient reported outcome measures (PROs) and disease activity, but also TNFi trough levels, calprotectin, HRQoL, physical functioning, work status, comorbidities and cardiovascular risk profile.

## Patients and methods

### Patients

In this cross-sectional, observational study a convenience sample of patients >18 years with IA (including RA, AS and PsA) currently under treatment with TNFi at the Department of Rheumatology, Sørlandet Hospital Kristiansand, were included from January 2016 to December 2017. The patients underwent assessment by a physician and nurse as part of standard care, and self-reported demographics and PROs in the computer system used for standard follow-up (GoTreatIT Rheuma®) [15]. Data on current and previous treatment were extracted from the GoTreatIt Rheuma database. The study was approved by the Norwegian Regional Committees for Medical and Health Research Ethics (2015/1196/REK midt). Written informed consent was obtained from each patient.

### Patient-reported outcome measures of disease activity

Patients self-reported on 0–100 mm visual analogue scales (VAS) global assessment of disease activity, pain, joint pain, back pain, morning stiffness and fatigue, patient’s satisfaction with their IA disease activity (much better/better/unchanged/worse/much worse) and Bath Ankylosing Spondylitis Disease Activity Index (BASDAI, range 0–10) [16] and Functional Index (BASFI, range 0–10) [17].

### Other measures of disease activity

Evaluator’s global assessment of disease activity was assessed together with 28 tender/swollen joint counts for the RA patients, 66/68 joint counts and Leeds Enthesitis Index [18] for the PsA patients and Maastricht Ankylosing Spondylitis Enthesitis Score [18] for the PsA and AS patients. Composite scores of disease activity were calculated: For the RA and PsA patients, 28-joint Disease Activity Score with ESR (DAS28-ESR) [1], DAS28 with CRP (DAS28-CRP) [1], Simplified Disease Activity Index (SDAI) [1], Clinical Disease Activity Index (CDAI) [1] and 28-joint Disease Activity index for PSoriatic Arthritis (DAPSA28) [19]. For PsA patients Minimal Disease Activity [20] (MDA) was also assessed, defined as fulfillment of 5 of 7 of the following criteria: 68 tender joint count ≤1, 66 swollen joint count ≤1, patient’s global assessment ≤20 mm, pain ≤15 mm, HAQ ≤0.5, Leeds Enthesitis Index ≤1 and Dermatology Life Quality Index (DLQI) [21] ≤5 (equivalent to no or small effect on patient’s life [21]). We used DLQI as a substitute for Psoriasis Activity and Severity Index (PASI), as we did not assess PASI in the study. For the AS and PsA patients Ankylosing Spondylitis Disease Activity Score (ASDAS) was assessed [16]. Remission was defined as DAS28-ESR<2.6 in RA [1], ASDAS<1.3 in AS [16] and MDA in PsA [16]. Erosions in hands and feet in RA and PsA patients (yes/no) were assessed on X-rays done no more than 2 years before the visit date. Bath Ankylosing Spondylitis Metrology Index [18] (BASMI) was assessed for the AS patients.

### Health-related quality of life

Patients’ self-reporting also included HRQoL, assessed by Short Form-36 V1 (SF-36), including the 8 scales on mental health, vitality, bodily pain, general health, social functioning, physical functioning, role physical and role emotional [22]. The Norwegian translation of SF-36 version 1 was used, which has been validated in Norwegian RA patients [23]. Physical Component Summary (PCS) and Mental Component Summary (MCS) were computed as outlined by Ware et al [24]. Comparison of SF-36 scale scores, PCS and MCS from the study patients with SF-36 from the Norwegian general population collected by Loge et al in in 1998 was also performed [25]. For the PsA patients we also assessed DLQI [21].

### Physical functioning

Stanford Health Assessment Questionnaire (HAQ, range 0–3) [26] was used for self-reporting of physical functioning. Further, patients reported their usual level of physical exercise (≥3 times a week/1-2 times a week/1-2 times a month/Do not exercise regularly/Cannot exercise due to disability or handicap).

### Work status

Patients reported their current employment within the following categories: full-time job, part-time job, sick leave, occupational rehabilitation, unemployed, disabled pensioner, pensioner, student, parental leave, part-time job/sick leave, part-time job/unemployed and part-time job/disabled pensioner. Patients also completed the Work Productivity and Activity Impairment-General Health (WPAI-GH) questionnaire, including the domains absenteeism (work time missed), presenteeism (impairment at work/reduced effectiveness at work), work productivity (overall work impairment) and activity impairment [27].

### Laboratory markers, including TNFi trough levels, neutralizing antibodies and calprotectin

C-reactive protein (CRP, mg/l), erythrocyte sedimentation rate (ESR, mm/h), leukocytes (G/l), uric acid (μmol/l), low-density lipoprotein (LDL, mmol/l), high-density lipoprotein (HDL, mmol/l), triglycerides (mmol/l), total cholesterol (mmol/l) and glycated hemoglobin (HbA1c, mmol/mol) were assessed as part of standard care. For analyses of TNFi trough levels, neutralizing antibodies and calprotectin, serum samples were stored at -80°C until further analyses were performed. CalproLab^TM^ (ALP) ELISA kits were used for assessment of calprotectin in serum, according to instructions by the manufacturer (Calpro AS, Norway) [28,29]. iLite^TM^ assay kits/ iLite^TM^ NAb assay kits were used for analyses of TNFi (infliximab, adalimumab, certolizumab, etanercept) trough levels and semi-quantitative determination of neutralizing antibodies to the different TNFi, using luciferase generated bioluminescence [30].

### Comorbidities

Patients reported to have, or to have a history of, 20 different comorbidities, including high blood pressure, angina pectoris, heart attack, heart failure, bypass operation, intermittent claudication, stroke/brain hemorrhage/TIA, asthma/bronchitis/other pulmonary disease, allergy/hay fever/eczema, chronic back pain, cancer, neurological disease, diabetes, hypothyroidism, mental illness, problems with alcohol or drugs, kidney disease, liver disease, stomach ulcer and anemia (yes/no). Presence of parents or brothers or sisters with cardiovascular disease before 65 years of age for women and 55 years for men, was also reported (yes/no).

### Cardiovascular risk

The Systematic Coronary Risk Evaluation project (SCORE) risk prediction algorithm was used to estimate the 10-year risk of a fatal cardiovascular event [31]. As recommended for Norway, we used the low risk version of SCORE [31]. The SCORE algorithm includes age, sex, smoking status, systolic blood pressure and total cholesterol/HDL ratio. Categorization into four SCORE groups were performed: Low risk (<1%), moderate risk (≥1% and <5%), high-risk (≥5% and <10%) and very high risk (≥10%).

### Statistics

Medians (25^th^-75^th^ percentiles) were calculated for non-normally and means (SD) for normally distributed data. Proportions were presented as n (%). Unadjusted comparisons across sex were performed with Mann-Whitney U-test, independent t-test, Fisher’s exact test or X^2^ test, as appropriate.

Age- and diagnosis-adjusted comparisons across sex were performed with General Linear Models (continuous outcomes), logistic regression (binary outcomes) and ordinal logistic regression (ordinal outcomes). Comparison of remission, TNFi trough levels and neutralizing antibodies across sex were additionally adjusted for current use of csDMARDs and BMI. Radar diagrams were made for visualization of SF-36 scale scores. All analyses were performed as completer analyses. No correction for multiple comparisons were done. A p-value <0.05 was considered significant. Sensitivity analyses with additional adjustment for disease duration and BMI, in addition to age and diagnosis, were also performed. Statistical tests were done using SPSS for Windows V.26.0.0.1.

## Results

We included a total of 305 patients with IA (120 RA, 108 AS and 77 PsA patients). One hundred and thirty-eight patients were women and 167 men (Table 1).

**Table 1:** Demographics.

	All patients (n = 305)	Men (n = 167)	Women (n = 138)
**Age (years), mean (SD),** **n available**	52.9 (12.8) n = 305	56.7 (13.2) n = 167	47.7 (11.4) n = 138
**Disease duration (years), mean (SD),** **n available**	12.5 (9.7) n = 305	12.1 (9.2) n = 167	13.0 (10.2) n = 138
**Currently smoking, n (%),** **n available**	44 (14.8) n = 298	25 (15.2) n = 164	19 (14.2) n = 134
**Uses snuff, n (%)** **n available**	19 (7.4) n = 258	14 (9.7) n = 144	5 (4.4) n = 114
**BMI, mean (SD),** **n available**	27.4 (9.5) n = 299	28.8 (12.1) n = 164	25.7 (4.4) n = 135
**Civil status (%)** **Single** **Married** **Cohabiter** **Separated** **Divorced** **Widower** **n available**	9.6% 61.8% 17.4% 1.1% 7.3% 2.8% n = 178	12.9% 61.3% 20.4% 1.1% 4.3% 0.0% n = 93	5.9% 62.4% 14.1% 1.2% 10.6% 5.9% n = 85
**Years of education, mean (SD)** **n available**	13.1 (0.2) n = 297	13.1 (3.4) n = 164	13.2 (3.3) n = 133
**Current TNFi, n (%)** **Adalimumab** **Etanercept** **Certolizumab** **Golimumab** **Infliximab** **n available**	51 (16.7) 92 (30.2) 66 (21.6) 15 (4.9) 81 (26.6) n = 305	33 (19.8) 51 (30.5) 37 (22.2) 10 (6.0) 36 (21.6) n = 167	18 (13.0) 41 (29.7) 29 (21.0) 5 (3.6) 45 (32.6) n = 138
**Previously bDMARD naïve, %,** **n available**	77% n = 305	86.8% n = 167	65.2% n = 138
**Concomitant csDMARDs, %,** **n available**	40.3% n = 305	32.9% n = 167	49.3% n = 138

BMI, body mass index; bDMARD, biologic disease-modifying anti-rheumatic drug; csDMARD, conventional synthetic DMARD; TNFi, tumor necrosis factor inhibitor.

The women were younger, had longer disease duration and lower BMI than the men. Similar proportions of men and women were smokers, whereas a higher proportion of men used snuff. The most common civil status was married, followed by cohabiter and single for men and cohabiter and divorced for women. Both men and women had a mean of 13 years of education. About three quarters of the patients were previously bionaïve with a higher proportion of men than women. Etanercept was the most commonly used TNFi, followed by infliximab, certolizumab, adalimumab and golimumab.

### Patient-reported outcome measures of disease activity

Women reported significantly more pain and joint pain than the men, but similar levels of back pain (adjusted analyses) and morning stiffness (Table 2).

**Table 2:** Comparison of disease activity measures, physical functioning, health-related quality of life and laboratory markers between men and women with inflammatory arthritis.

	All patients (n = 305)	Men (n = 167)	Women (n = 138)	p-value, unadjusted	p-value, adjusted*
**Pain (0–100 VAS),** **median (IQR)**	27 (10, 50)	25 (9, 47)	30 (15, 55)	0.05	0.008
**Joint pain (0–100 VAS),** **median (IQR)**	26 (10, 48)	8 (2, 25)	14 (3, 29)	0.14	0.03
**Back pain (0–100 VAS),** **median (IQR)**	24 (6, 50)	23 (5, 46)	24 (5, 56)	0.43	0.095
**Morning stiffness (h),** **median (IQR)**	0.5 (0.2, 1.0)	0.5 (0.1, 1.0)	0.5 (0.2, 1.4)	0.41	0.37
**Fatigue (0–100 VAS),** **median (IQR)**	36 (14, 66)	30 (9, 63)	39 (18, 74)	0.06	0.03
**Patients’ global (0–100 VAS), median (IQR)**	27 (12, 50)	10 (25, 50)	15 (29, 50)	0.16	0.06
**Investigator’ global (0–100 VAS), median (IQR)**	4 (0, 10)	4 (0, 10)	5 (0, 13)	0.23	0.61
**Patient’s satisfaction** ** • Much better** ** • Better** ** • Unchanged** ** • Worse** ** • - Much worse**	33.9% 37.8% 11.7% 14.8% 1.8%	38.2% 33.8% 10.8% 15.3% 1.9%	28.6% 42.9% 12.7% 14.3% 1.6%	0.44	0.36
**BASDAI, median (IQR)**	2.6 (1.0, 4.6)	2.8 (1.0, 4.4)	2.5 (1.1, 5.7)	0.47	0.35
**BASFI, median (IQR)**	2.2 (0.6, 4.0)	2.3 (0.5, 4.0)	1.6 (0.8, 4.1)	0.77	0.86
**MASES, median (IQR)**	2 (0, 5)	1 (0, 4)	4 (2, 6)	<0.001	<0.001
**% of patients with MASES >0,**	69%	56%	86%	<0.001	<0.001
**28 tender joint count,** **median (IQR)**	0 (0, 2)	0 (0, 1)	0 (0, 2)	0.31	0.22
**28 swollen joint count,** **median (IQR)**	0 (0, 0)	0 (0, 0)	0 (0, 0.5)	0.12	0.43
**68 tender joint count,** **median (IQR)**	1 (0, 4)	1 (0, 3)	1 (0, 4)	0.85	0.76
**66 swollen joint count,** **median (IQR)**	0 (0, 0)	0 (0, 0)	0 (0, 0)	0.88	0.90
**DAS28ESR, mean (SD)**	2.6 (1.1)	2.3 (1.1)	2.8 (1.0)	0.001	0.001
**DAS28CRP, mean (SD)**	2.3 (0.9)	2.3 (0.9)	2.4 (0.9)	0.34	0.37
**CDAI, median (IQR)**	4.6 (2.0, 8.1)	4.5 (1.4, 7.5)	5.0 (2.2, 9.3)	0.08	0.11
**SDAI, median (IQR)**	5.0 (2.1, 8.8)	4.6 (1.7, 8.1)	5.3 (2.7, 9.4)	0.11	0.22
**DAPSA28, median (IQR)**	8.5 (3.4, 14.1)	7.3 (2.5, 12.9)	9.4 (4.4, 16.6)	0.06	0.06
**ASDAS, median (IQR)**	1.6 (1.0, 2.4)	1.6 (0.9, 2.3)	1.7 (1.1, 2.5)	0.42	0.24
**MDA, n (%)**	52 (77.6)	36 (80.0)	16 (72.7)	0.50	0.82
**Patients in remission** ^1^ **, n (%)**	154 (52.6)	94 (58.8)	60 (45.1)	0.02	0.009 (0.008^§^)
**Erosions in hands or feet, %**	59.3%	56.8%	61.7%	0.50	0.39
**SF-36 Mental Health, mean (SD)**	75.0 (17.0)	74.5 (18.1)	75.7 (15.6)	0.94	0.81
**SF-36 Vitality, mean (SD)**	44.6 (22.4)	46.5 (22.9)	42.4 (21.8)	0.17	0.04
**SF-36 Bodily Pain, mean (SD)**	55.1 (23.9)	57.1 (23.7)	52.5 (23.9)	0.11	0.07
**SF-36 General Health, mean (SD)**	52.3 (20.6)	52.7 (20.7)	52.0 (20.6)	0.88	0.53
**SF-36 Social Functioning,** **mean (SD)**	71.5 (24.5)	73.6 (24.5)	68.9 (24.2)	0.098	0.03
**SF-36 Functioning Physical, mean (SD)**	68.0 (22.9)	71.0 (22.6)	64.2 (22.9)	0.02	0.07
**SF-36 Role Physical, mean (SD)**	41.1 (42.1)	44.5 (42.5)	36.7 (41.4)	0.14	0.09
**SF-36 Role Emotional, mean (SD)**	67.1 (40.5)	68.5 (40.0)	65.2 (41.3)	0.51	0.34
**SF-36 MCS, mean (SD)**	47.4 (11.3)	48.1 (11.9)	48.1 (10.5)	0.99	0.39
**SF-36 PCS, mean (SD)**	38.4 (11.0)	38.4 (11.0)	35.5 (10.7)	0.03	0.06
**HAQ, median (IQR)**	0.5 (0.1, 1.0)	0.4 (0.0, 0.9)	0.6 (0.3, 1.3)	0.002	0.01
**Physical exercise:** **≥3 times/week** **1–2 times/week** **1–2 times/month** **Do not exercise regularly** **Cannot exercise due to disability or handicap**	28.7% 31.3% 3.6% 32.0% 4.4%	30.0% 32.0% 3.3% 29.3% 5.3%	27.2% 30.4% 4.0% 35.2% 3.2%	0.78	0.73
**ESR (mm/h), median (IQR)**	9 (4, 17)	6 (3, 13)	14 (8, 19)	<0.001	0.04
**CRP (mg/L), median (IQR)**	2 (1, 4)	1 (1, 3)	2 (1, 4)	0.54	0.42
**Serum calprotectin (ng/ml), median (IQR)**	816 (585, 1149)	758 (580, 1116)	848 (596, 1160)	0.33	0.63
**Leukocytes (G/l), median (IQR)**	6.7 (5.6, 8.2)	6.5 (5.6, 7.8)	7.2 (5.8, 8.4)	0.05	0.17
**Uric acid (μmol/l), mean (SD)**	306 (80)	332 (79)	276 (71)	<0.001	<0.001
**Infliximab trough serum level, mg/l, median (IQR)**	1.6 (1.0–5.2)	2.4 (1.0, 6.9)	1.3 (0.9, 4.9)	0.33	0.99 (0.82^§^)
**Adalimumab trough serum level, mg/l, median (IQR)**	4.4 (1.8–7.7)	4.1 (1.7, 7.5)	5.1 (1.8, 11.4)	0.36	0.11 (0.19^§^)
**Certolizumab trough serum level ≥20 mg/l, %**	89%	92%	86%	0.69	0.45 (0.35^§^)
**Etanercept trough serum level, mg/L, median (IQR)**	2.0 (1.7, 8.3)	3.7 (1.7, 8.5)	1.9 (1.7, 6.1)	0.47	0.99 (0.94^§^)
**% of patients with TNFi trough level above lower reference value** ^**2**^	66%	68%	63	0.41	0.43 (0.27^§^)
**% of patients with neutralizing antibodies to TNFi**	3.9%	2.6%	5.4%	0.36	0.24 (0.13^§^)

*Adjusted for age and diagnosis. ^1^Remission defined by DAS28<2.6 for RA patients, ASDAS<1.3 for AS patients and MDA for PsA patients; ^2^For infliximab ≥3μg/ml, adalimumab ≥5 μg/ml, certolizumab ≥20 μg/ml, etanercept ≥1.5 μg/ml; ^§^adjusted for age, diagnosis, current use of csDMARDs and BMI; ASDAS, Ankylosing Spondylitis Disease Activity Score; BASDAI, Bath Ankylosing Spondylitis Disease Activity Index; BASFI, Bath Ankylosing Spondylitis Functional Index; BASMI, Bath Ankylosing Spondylitis Meteorology Index; DAPSA28, 28-joint Disease Activity index for PSoriatic Arthritis; DAS28-CRP, 28-joint Disease Activity Score with CRP; DAS28-ESR, DAS28 with ESR; CDAI, Clinical Disease Activity Index; CRP, C-reactive protein; ESR, erythrocyte sedimentation rate; HAQ, Stanford Health Assessment Questionnaire; IQR, interquartile range; MASES, Maastricht Ankylosing Spondylitis Enthesitis Score; SDAI, Simplified Disease Activity Index; MCS, Mental Component Summary; MDA, minimal disease activity; PCS, Physical Component Summary; SD, Standard Deviation; SF-36, Short Form-36; TNFi, Tumor Necrosis Factor inhibitors

Fatigue was also significantly worse in women than men. BASDAI and BASFI were similar across the sexes. As to patients’ satisfaction with their IA disease activity, 72% of the patients reported their condition to be much better or better, 12% unchanged and 16% worse or much worse. (Table 2). Patient satisfaction was similar across the sexes.

### Other measures of disease activity

Composite scores of disease activity including DAS28CRP, DAS28ESR, CDAI, SDAI, DAPSA28 and ASDAS were all numerically higher in women than men, but only DAS28ESR was significantly worse in women. A significantly higher proportion of men than women were in remission in unadjusted (p = 0.02) as well as age- and diagnosis-adjusted analyses (p = 0.009, Table 2). Also with additional adjustment for current use of csDMARDs and BMI these differences were significant (p = 0.008). Investigator’s global assessment of disease activity was similar across the sexes, whereas the enthesitis measure MASES was significantly higher in women. A similar proportion of men and women had erosions in hands and feet. BASMI was similar across the sexes.

### Health-related quality of life

**Fig 1:**
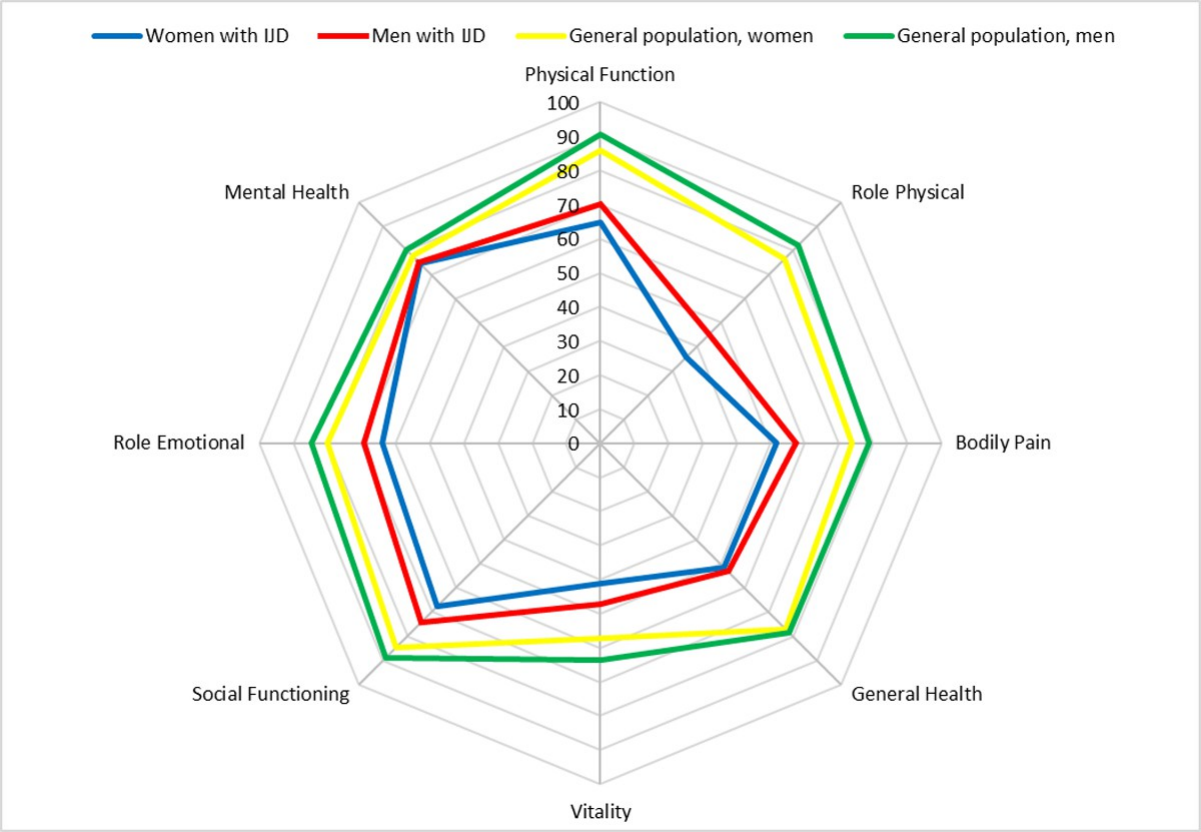
SF-36 scale scores (estimated marginal means) for men and women with inflammatory arthritis (adjusted for age and diagnosis) and for men and women from the general Norwegian population (adjusted for age).

In adjusted analyses, women had significantly worse scores on the SF-36 vitality and social functioning scales than men, but similar SF-36 mental health, bodily pain, general health, physical functioning, role physical and role emotional scale scores, although several of these measures showed a trend versus worse values in women (Table 2). SF-36 PCS was significantly worse in women in unadjusted (p = 0.03) but not adjusted analyses (p = 0.06), whereas SF-36 MCS was similar across the sexes. In Fig 1 sex-specific SF-36 scale scores adjusted for age and diagnosis are visualized in a radar diagram, together with age-adjusted sex-specific scale scores from the Norwegian general population.

### Physical functioning

Physical functioning measured by HAQ, was significantly better in men than in women. Both sexes reported similar levels of physical exercise. A majority exercised 1–2 times a week or 3 or more times a week. About a third did not exercise regularly and about 4% did not exercise due to disability or handicap.

### Work status

Work status differed significantly across the sexes (Table 3).

**Table 3:** Work status.

	All patients (n = 305)	Men (n = 167)	Women (n = 138)	p-value, unadjusted	p-value, adjusted*
**Persons <67 years currently in full-time work, n (%), n available**	88 (33.8) n = 260	70 (47.9) n = 146	18 (15.8) n = 114	<0.001	<0.001
**Persons <67 years currently in part-time work, n (%), n available**	71 (27.3) n = 260	23 (15.8) n = 146	48 (42.1) n = 114	<0.001	<0.001
**Work status, all ages, n (%)** **Full-time work** **Part-time work** **Sick leave** **Occupational rehabilitation** **Unemployed** **Disabled pensioner** **Pensioner** **Student** **Parental leave** **Part-time job/sick leave** **Part-time job/unemployed** **Part-time job/ disabled pensioner** **n available**	92 (31.0) 32 (10.8) 8 (2.7) 15 (5.1) 5 (1.7) 60 (20.2) 38 (12.8) 5 (1.7) 2 (0.6) 12 (4.0) 2 (0.7) 26 (8.8) n = 297	73 (44.8) 9 (5.5) 4 (2.5) 9 (5.5) 3 (1.8) 27 (16.6) 18 (11) 4 (2.5) 1 (0.6) 3 (1.8) 2 (1.2) 10 (6.1) n = 163	19 (14.2) 23 (17.2) 4 (3.0) 6 (4.5) 2 (1.5) 33 (24.6) 20 (14.9) 1 (0.7) 1 (0.7) 9 (6.7) 0 (0) 16 (11.9) n = 134	<0.001	0.01
**WPAI-GH, % of absenteeism,** **mean (SD), n available**	7.7 (20.1) n = 144	4.6 (14.8) n = 88	12.4 (25.9) n = 56	0.046	0.02
**WPAI-GH, % of presenteeism,** **mean (SD), n available**	26.0 (22.8) n = 141	24.3 (22.9) n = 88	28.9 (22.5) n = 53	0.25	0.20
**WPAI-GH, % of work productivity loss, mean (SD), n available**	28.8 (26.0) n = 141	26.8 (25.8) n = 88	32.3 (26.2) n = 53	0.23	0.20
**WPAI-GH, % of activity impairment, mean (SD), n available**	35.2 (25.5) n = 258	32.7 (25.4) n = 143	38.3 (25.3) n = 115	0.08	0.04

*adjusted for age and diagnosis; WPAI- GH, Work Productivity and Activity Impairment-General Health; SD, Standard Deviation.

Significantly higher proportions of men were currently in full-time work and women in part-time work. There were no clear differences in presenteeism and work productivity loss across the sexes, whereas absenteeism and activity impairment were worse in women (Table 3).

### Laboratory markers, including TNFi trough levels, neutralizing antibodies and calprotectin

Women had significantly higher ESR than the men, but similar CRP, calprotectin and leukocytes in unadjusted, as well as age and diagnosis adjusted analyses (Table 2). Women and men had similar serum trough levels of infliximab, adalimumab, certolizumab and etanercept (Table 2). Similar proportions of men and women had TNFi trough levels above the lower reference value. Eleven (4%) of the patients had neutralizing antibodies to TNFi, thereof 9 to infliximab, 1 to certolizumab and 1 to etanercept. Similar proportions of men and women had neutralizing antibodies.

### Comorbidities

Information on self-reported comorbidities was available in 263 (144 men and 119 women) of the 305 patients. About a fourth of the patients reported no comorbidities. Further, about a third of the patients had 1 comorbidity, about a fifth 2 comorbidities and also about a fifth 3–4 comorbidities. The most common comorbidities were chronic back pain, high blood pressure and allergy/hay fever/eczema, followed by asthma/bronchitis/other pulmonary disease, stomach ulcer, mental illness, diabetes and hypothyroidism. A similar total number of comorbidities were seen across the sexes. Consistently in unadjusted and adjusted analyses, mental illness was more frequently reported by men and hypothyroidism by women.

### Cardiovascular risk

Women had more frequently parents or brothers and sisters with coronary heart disease in young age (Table 4). Men had significantly higher systolic and diastolic blood pressure.

**Table 4:** Cardiovascular risk profile.

	All patients (n = 305)	Men (n = 167)	Women (n = 138)	p-value, unadjusted	p-value, adjusted*
**Systolic blood pressure (mm Hg), mean (SD)**	131 (15)	134 (15)	128 (15)	0.002	0.002
**Diastolic blood pressure (mm Hg), mean (SD)**	81 (9)	82 (9)	80 (8)	0.01	0.045
**LDL (mmol/l),** **mean (SD)**	3.3 (1.0)	3.3 (1.0)	3.4 (1.0)	0.54	0.59
**HDL (mmol/l),** **mean (SD)**	1.5 (0.5)	1.3 (0.4)	1.8 (0.5)	<0.001	<0.001
**Triglycerides (mmol/l),** **mean (SD)**	1.6 (1.0)	1.8 (1.2)	1.4 (0.7)	<0.001	0.001
**Total cholesterol (mmol/l),** **mean (SD)**	5.1 (1.1)	4.9 (1.1)	5.3 (1.1)	0.003	0.007
**HbA1c (mmol/mol),** **mean (SD)**	5.6 (0.7)	5.6 (0.8)	5.6 (0.5)	0.64	0.19
**Parents, brothers or sisters with young-age coronary heart disease** ^**1**^ **, n (%)**	59 (22.4)	24 (16.7)	35 (29.4)	0.01	0.03
**10-year risk of a fatal cardiovascular event,** **median (IQR)**	0.9 (0.4, 2.1)	1.2 (0.5, 2.5)	0.7 (0.3, 1.6)	<0.001	<0.001
SCORE** **Low risk (<1%), n (%)** **Moderate risk (≥1 and <5), n (%)** **High risk (≥5 and <10%) and very high risk (>10%), n (%)**	98 (50.8) 84 (43.5) 11 (5.7)	46 (42.2) 54 (49.5) 9 (8.3)	52 (61.9) 30 (35.7) 2 (2.4)	0.01	<0.001

*Adjusted for age and diagnosis; HbA1c, glycated hemoglobin; HDL, high-density lipoprotein; IQR, interquartile range; LDL, low-density lipoprotein; SCORE, The Systematic Coronary Risk Evaluation project risk prediction algorithm, SD, Standard Deviation.

LDL was similar across the sexes, HDL and total cholesterol higher in women and triglycerides higher in men. HbA1c was similar across the sexes. The 10-year risk of a fatal cardiovascular event according to SCORE was significantly higher in men. A higher proportion of men than women had high/very high as well as moderate risk of a 10-year fatal cardiovascular event.

### Sensitivity analyses

Sensitivity analyses with adjustment not only for age and diagnosis, but also for disease duration and BMI, did not change the significance of the findings, except for change from similar to significantly worse scores in women for patient global (p = 0.03), SF-36 bodily pain (p = 0.02), SF-36 physical functioning (p = 0.01) and SF-36 PCS (p = 0.01). For comorbidities, when also adjusting for BMI and disease duration in addition to age and diagnosis, self-reported mental illness changed from being significantly more frequently reported by men to being similar across the sexes (p = 0.05).

## Discussion

In this observational, cross-sectional study of 305 patients with inflammatory arthritis treated with TNFi, we found important differences in disease burden between men and women. A significantly lower proportion of women than men were in remission according to disease-specific composite scores. Women reported significantly more pain, joint pain and fatigue and had higher MASES. Furthermore, women had significantly worse SF-36 vitality and social functioning scale scores than men. Both sexes had worse SF-36 scale scores, PCS and MCS compared with the Norwegian general population [25]. Physical functioning (HAQ) was better in men. Still, both sexes reported similar levels of physical exercise. Women were more often in part-time work and reported more absenteeism (work time missed) and activity impairment than the men. Proportions of patients with erosions in hands or feet, TNFi trough levels, neutralizing antibodies and calprotectin were similar across the sexes. A similar total number of comorbidities was seen for men and women. Hypothyroidism was more frequent in women. Men had a less favorable cardiovascular risk profile than women and a higher 10-year risk of a fatal cardiovascular event.

In line with previous studies, is the finding of less women than men being in remission [7,11,12]. This finding was statistically significant in analyses adjusted for age and sex, in analyses additionally adjusted for csDMARD use and BMI as well as in sensitivity analyses, and in spite of all patients being currently under treatment with TNFi. All composite scores of disease activity were numerically higher in women, although only DAS28-ESR reached statistical significance. ESR was higher in women (which has also been shown in the general population) and may explain the differences found between DAS28ESR and DAS28-CRP levels. Of note, in early phase clinical trials there is a persistent underrepresentation of women, despite accumulating evidence in support of sex-based differences in immune responses and prevalence of autoimmune diseases [32,33]. Proportions of patients with TNFi trough levels above the lower reference values and proportions of patients with neutralizing antibodies to TNFi, were similar for men and women in unadjusted analyses, analyses adjusted for age, sex, current csDMARD comedication and BMI as well as in sensitivity analyses. In accordance with other studies, most neutralizing antibodies were found against infliximab [34]. We had the opportunity to analyze calprotectin in serum and found similar levels of calprotectin across the sexes. Although not routinely measured in most rheumatology clinics, calprotectin is found to be a promising marker of inflammation in patients with IA [35].

We found worse pain, joint pain, fatigue and MASES in women vs. men, which is in line with other studies [7,9,11–14]. Interestingly, animal studies have shown that chronic pain in female mice, unlike in male mice, may additionally be mediated by adaptive immune cells, indicating that male mice cannot be used as proxies for females in pain research [36]. Psychological and social factors may also contribute to sex differences in pain [37]. Further, a lower proportion of women than men being in remission may also be of impact.

All SF-36 scale scores in our population of patients with IA were worse than in the Norwegian general population, which is in accordance with previous findings [38]. As to sex differences, all SF-36 scale scores were numerically worse in women, like also seen in the general population [25]. Women had significantly worse vitality and social functioning scale scores than men. Similar findings of worse vitality in women have previously been reported in a study of 271 Norwegian PsA patients [39]. Unlike our study, only adults aged 18–45 years were included in that study and no difference in social functioning across the sexes was found [39]. Our findings were confirmed in sensitivity analyses, in which also the SF-36 bodily pain and physical functioning scale scores were significantly worse in women.

Compared with the general population, we found SF-36 PCS to be more impaired than SF-36 MCS, which is in line with findings from the observational NOR-DMARD study on RA and PsA patients [38]. SF-36 PCS was somewhat better in our study than in the NOR-DMARD study, which included patients between 2000 and 2012, possibly reflecting the improved treatment options in 2016–2017 when our study was conducted [38]. Women had worse SF-36 PCS than men in unadjusted analyses as well as analyses adjusted for age, diagnosis, BMI and disease duration, but not when adjusting only for age and diagnosis. Women with IA have also in previous studies been found to have worse SF-36 PCS than men [9].

HAQ was worse in women, which is in line with previous reports [8,39,40]. Still, this did not seem to impact upon levels of physical exercise, which was similarly reported by the sexes. Overall, 61% of persons aged 66 and below were working. The common retirement age in Norway is 67 years, but some employees have a collective agreement of early retirement between the age of 62 and 67. In the general Norwegian population about 68% of men and 67% of women aged 18–74 years are currently working [41]. Significantly higher proportions of men were currently in full-time work and women in part-time work, similar to what is seen in the Norwegian general population [41]. Women experienced more absenteeism and activity impairment than men. A contributing factor to this might be the higher level of fatigue in the women, as fatigue has been found to contribute to work productivity impairment in patients with IA [42].

Comorbidities may impact upon treatment, as patients with specific comorbidities or multimorbidity may be more difficult to treat and less often achieve remission/the agreed treatment target [4,43]. No differences in number of comorbidities were seen across the sexes, neither in unadjusted analyses, nor in age-adjusted analyses or sensitivity analyses. Consistently across unadjusted and adjusted analyses, men more frequently self-reported mental illness and women more often hypothyroidism. However, sensitivity analyses did not confirm the finding of more frequent mental illness in men. A recent study has raised focus on the association between arthritis and autoimmune thyroid disorders and suggested regularly thyroid function follow-up particularly in women at high risk [44]. Studies have also raised focus on mental disorders like depression and anxiety, which may occur more often in patients with IA, with negative impact upon treatment outcomes [45].

Patients with IA have an increased risk of cardiovascular disease compared with the general population [46,47]. However, studies have shown that targeting inflammation with TNFi may decrease this risk [48]. In our study where all patients were under treatment with TNFi, we found a higher estimated 10-year risk of a fatal cardiovascular event in men than in women. Men had higher systolic and diastolic blood pressure, lower HDL and higher triglycerides, whereas level of LDL was similar across the sexes. Of note, at any given age, estimated 10-year risk of fatal cardiovascular disease according to SCORE is lower for women than men [49].

Limitations of the study include missing data for some of the outcomes, like often may be the case in observational studies. However, the proportion of missing data were in general acceptable, with e.g. about 15% missing for the different SF-36 measures. Further, as this was an observational study, no conclusions on causality may be drawn. We used disease-specific composite scores for assessment of remission, that is, DAS28<2.6 in RA, MDA in PsA and ASDAS<1.3 in AS patients. Still, a limitation of the study may be that we included different types of IA patients. However, we found consistent results also when adjusting for type of IA, as well as in sensitivity analyses.

The main strength of the study is that, to our knowledge, this is the study on sex differences in IA assessing the most comprehensive panel of health aspects, including not only PROs like HRQoL and pain measures, but also disease activity, TNFi trough levels, neutralizing antibodies, calprotectin, work status, physical functioning, comorbidities and cardiovascular risk. We believe that our study may raise awareness to the sex gap in reporting of clinical data, where more focus on women is warranted, to ensure better tailoring of treatment and follow-up also for women.

In conclusion, in this observational study on patients with IA treated with TNFi, less women than men were in a state of remission. Further, women had worse pain, joint pain, fatigue and enthesitis scores as well as worse SF-36 vitality and social functioning scale scores. TNFi trough levels, neutralizing antibodies and calprotectin were similar across the sexes. More men were in full-time and more women in part-time work. Women experienced more absenteeism and activity impairment. A higher proportion of women had a history of hypothyroidism. Men had a less favorable cardiovascular risk profile and a higher 10-year risk of a fatal cardiovascular event. Our study highlights that clinicians should be aware of sex differences when interpreting clinical outcomes and treatment responses in IA patients, especially when it comes to differences in perception of symptoms, e.g. pain and fatigue.
